# The impact of image degradation and temporal dynamics on sustained attention

**DOI:** 10.1167/jov.22.4.8

**Published:** 2022-03-17

**Authors:** Julia M. Brau, Alexander Sugarman, David Rothlein, Joseph DeGutis, Michael Esterman, Francesca C. Fortenbaugh

**Affiliations:** 1 Translational Research Center for TBI and Stress Disorders (TRACTS), VA Boston Healthcare System, Boston, MA, USA; 2 Boston Attention and Learning Lab (BALLAB), VA Boston Healthcare System, Boston, MA, USA; 3 National Center for PTSD, VA Boston Healthcare System, Boston, MA, USA; 4 Department of Psychiatry, Harvard Medical School, Cambridge, MA, USA; 5 Department of Psychiatry, Boston University School of Medicine, Boston, MA, USA; 6 National Center for PTSD, VA Boston Healthcare System, Boston, MA, USA; 7 Translational Research Center for TBI and Stress Disorders (TRACTS), VA Boston Healthcare System, Boston, MA, USA; 8 Boston Attention and Learning Lab (BALLAB), VA Boston Healthcare System, Boston, MA, USA; 9 Department of Psychiatry, Boston University School of Medicine, Boston, MA, USA

**Keywords:** sustained attention, continuous performance task, contrast sensitivity, resolution

## Abstract

Many clinical populations that have sustained attention deficits also have visual deficits. Therefore, it is necessary to understand how the quality of visual input and different forms of image degradation can contribute to worse performance on sustained attention tasks, particularly those with dynamic and complex visual stimuli. This study investigated the impact of image degradation on an adapted version of the gradual-onset continuous performance task (gradCPT), where participants must discriminate between gradually fading city and mountain scenes. Thirty-six normal-vision participants completed the task, which featured two blocks of six resolution and contrast levels. Subjects either completed a version with gradually fading or static image presentations. The results show decreases in image resolution impair performance under both types of temporal dynamics, whereas performance is only impaired under gradual temporal dynamics for decreases in image contrast. Image similarity analyses showed that performance has a higher association with an observer's ability to gather an image's global spatial layout (i.e. gist) than local variations in pixel luminance, particularly under gradual image presentation. This work suggests that gradually fading attention paradigms are sensitive to deficits in primary visual function, potentially leading to these issues being misinterpreted as attentional failures.

## Introduction

Sustained attention, the effortful process of focusing on tasks or goals while suppressing distracting information over prolonged periods of time, is critical for our daily interactions, whether it be maintaining focus while driving, attending to a lecture, or navigating through a crowded area (Chun, Golomb, & Turk-Browne, 2011; Fortenbaugh, DeGutis, & Esterman, 2017; Langner & Eickhoff, 2013; Sarter, Givens, & Bruno, 2001). Early experimental paradigms used to study sustained attention, including declines in performance with time on task typically referred to as the vigilance decrement, assessed performance capabilities on monotonous tasks that span from 30 minutes to hours (Mackworth, 1948). However, the longer time frames of many sustained attention paradigms have also been argued to have limited clinical practicality, as children and individuals with brain injury may not have the ability to engage with tasks of such long durations (Nuechterlein, Parasuraman, & Jiang, 1983). In response, paradigms have been developed to assess overall decrements in shorter time periods by increasing task difficulty through various types of paradigm modifications. These modifications have included increasing the rate of individual trial events that must be completed continuously without breaks over a period of time (Ballard, 1996), incorporation of response inhibition with responses to frequent non-targets and withholding of response to rare targets (Conners, Staff, Connelly, Campbell, MacLean, & Barnes, 2000; Robertson, Manly, Andrade, Baddeley, & Yiend, 1997), and increasing stimulus complexity or degrading the quality of the images to impede perceptual discrimination (Nuechterlein et al., 1983).

Of particular relevance for the current study, is the impact that stimulus complexity has on perceptual and behavioral responses due to interactions between attention and visual processing (Roebuck, Freigang, & Barry., 2016). Visual stimuli in continuous performance tasks (CPTs), such as the Sustained Attention to Response Task (SART), include abrupt stimulus onsets that exogenously re-orient participants (MacLean, Aichele, Bridwell, Mangun, Wokciulik, & Saron, 2009; Robertson et al., 1997; Yantis & Jonides, 1984). The gradual-onset continuous performance task (gradCPT), on the other hand, uses complex visual stimuli that fade from one to the next in rapid succession (Esterman, Noonan, Rosenberg, & Degutis, 2013). The stimuli used in the gradCPT consist of complex scene images, whereas the gradual fading of the task reduces image saliency across trials as any one image is only fully clear for 50 ms at a time. Beyond this time window, the scenes are overlaid with the former and subsequent scenes, reducing the clarity of the trial scene image throughout the task. These task parameters combined with the lack of abrupt stimulus onsets are thought to increase the difficulty of this task not only by eliminating exogenous cues from abrupt onsets but also by reducing signal salience, consistent with previous literature (Nuechterlein et al., 1983; Parasuraman, de Visser, Clarke, McGarry, Hussey, Shaw, & Thompson, 2009; Temple, Warm, Dember, Jones, LaGrange, & Matthews, 2000). Gradual fading of stimuli may require accumulated processing of stimulus information over time, providing room for more top-down, predictive processes to affect performance. Studies have shown that performance on the gradCPT can be used to characterize moment-to-moment fluctuations in sustained attention and has shown sensitivity to discriminate between varying states of task engagement during an experiment while exhibiting vigilance decrements in as little as 8 minutes time (Esterman et al., 2013; Esterman, Reagan, Liu, Turner, & DeGutis, 2014; Esterman, Rosenberg, & Noonan, 2014; Fortenbaugh, Rothlein, McGlinchey, Degutis, & Esterman, 2018; Kucyi, Esterman, Riley, & Valera, 2016; Kucyi, Daitch, Raccah, Zhao, Zhang, Esterman, Zeineh, Halpern, Zhang, Zhang, & Parvizi, 2020). Other lines of work have also demonstrated that increasing visual difficulty by reducing stimulus saliency generates vigilance decrements over shorter time durations. These studies have shown that vigilance decrements can be obtained in under 8 minutes through projector lens defocusing overlaid with noise masks (Nuechterlein et al., 1983), low-pass filtering with pixelated noise (Nuechterlein et al., 1983; Parasuraman et al., 2009), or reducing contrast in cluttered displays of high frequency information (Temple et al., 2000). Although there is a clear link between stimulus salience and time-on-task decrements in the literature with decreased stimulus saliency relating to greater vigilance decrements, parametric investigations of image degradation from blur and contrast manipulations using within-subject designs have not been done. Thus, previous research has demonstrated that making an image harder to see decreases performance both overall and over time but work to date has not addressed the question of whether how one makes an image harder to see differentially impacts performance for a fixed set of stimuli used in CPT tasks.

Although manipulating image complexity or saliency has been used to hasten performance declines with time on task, it is important to note that in clinical settings overall performance measures, such as accuracy rates or reaction time variability, are used as important clinical indicators of sustained attention ability (Auerbach, Kim, Chango, Spiro, Cha, Gold, Esterman, & Nock, 2014; Conners et al., 2000; DeGutis, Esterman, McCulloch, Rosenblatt, Milberg, & McGlinchey, 2015; Esterman, Fortenbaugh, Pierce, Fonda, DeGutis, Milberg, & McGlinchey, 2019; Park, Aul, DeGutis, Lo, Poole, McGlinchey, Bean, Leritz, & Esterman, 2021). Manipulating task difficulty through image complexity or image degradation to induce time-on-task decrements more readily raises the question of whether overall measures of ability continue to reflect sustained attention capabilities on these tasks or may be confounded with visual processing limitations. Although CPTs are used to measure sustained attention in many clinical populations with higher rates of primary visual function deficits, image quality may not always differentially impact performance in these populations based on the nature of the task. For example, Berardi, Parasuraman, and Haxby (2001) showed that image degradation using the defocusing approach developed by Nuechterlein et al. (1983) on a digit discrimination CPT reduced performance overall, but no main effect of age or interaction with age group was found for overall performance or vigilance decrements across young, middle-aged, and older adult age groups. Conversely, the gradCPT has exhibited robust age-related effects across the lifespan with performance declines observed in older adults (Fortenbaugh, DeGutis, Germine, Wilmer, Grosso, Russo, & Esterman, 2015). Age-related declines in visual function are also well-established (Owsley, 2011). Presbyopia, which reduces resolving power for near objects due to a loss of elasticity in the lens of the eye, has a current estimated prevalence of over 60% for individuals over 50 years of age, with roughly 45% of presbyopia cases uncorrected globally (Fricke, Tahhan, Resnikoff, Papas, Burnett, Ho, Naduvilath, & Naidoo, 2018). Although performance declines have been observed under degraded simple or object-based stimuli (Nuechterlein et al., 1983; Parasuraman et al., 2009; Temple et al., 2000), such image manipulations have not been studied in paradigms using more complex scene-based stimuli with documented age-related declines in performance.

It is therefore important to consider whether perceptual discrimination of complex, naturalistic scenes is more susceptible to certain alterations in image quality, such as changes in contrast and resolving power, that may commonly accompany certain vision-related conditions. Many clinical disorders present with changes in both contrast and acuity, such as glaucoma (Richman, Lorenzana, Lankaranian, Dugar, Mayer, Wizov, & Spaeth, 2010) and cataracts (Datta, Foss, Grainge, Gregson, Zaman, Masud, Osborn, & Harwood, 2008; Shandiz, Derakhshan, Daneshyar, Azimi, Moghaddam, Yekta, Yazdi, & Esmaily, 2011), which predominantly impact older adults, as well as traumatic brain injury (TBI; Bulson, Jun, & Hayes, 2012; Fortenbaugh, Gustafson, Fonda, Fortier, Milberg, & McGlinchey, 2021; Spiegel, Reynaud, Ruiz, Laguë-Beauvais, Hess, & Farivar, 2016). However, other conditions differentially impact one of these domains more prominently than the other. For example, depression has mainly been shown to be associated with decreased contrast sensitivity (Bubl, Tebartz Van Elst, Gondan, Ebert, & Greenlee, 2009; Fam, Rush, Haaland, Barbier, & Luu, 2013), whereas presbyopia is associated with decreased acuity at close ranges (Gupta, Wolffsohn, & Naroo, 2009; McDonnell, Lee, Spritzer, Lindblad, & Hays, 2003). In addition, several studies have suggested comorbidities between attention deficit hyperactivity disorder (ADHD) and vision problems characterized by both refractive and nonrefractive errors (Akmatov, Ermakova, & Bätzing, 2019; DeCarlo, Swanson, McGwin, Visscher, & Owsley, 2016; Granet, 2014; Reimelt, Wolff, Hölling, Mogwitz, Ehrlich, & Roessner, 2018; Su, Tsai, Tsai, & Tsai, 2019). Finally, image clarity can be impacted by environmental setting, with stimulus contrast independently impacted by glare on a computer screen (Paulsson & Sjöstrand, 1980; Rodriguez, Yamin Garretón, & Pattini, 2016). As different dimensions of visual spatial processing can be impacted in several clinical populations for whom sustained attention ability is often clinically assessed, one goal of the current study was to independently assess the impact of contrast changes and image resolution on performance in a continuous performance task.

In addition to parametrically assessing the relationship between stimulus salience and sustained attention performance, the current study sought to examine the relationship among image degradation type, image confusability, and behavioral performance. Notably, recent work on attention and stimulus features has demonstrated that the fidelity of image representations is not static or wholly tied to retinal and optical factors but also fluctuates as a function of attentional states. Specifically, optimal attentional states have been associated with increased fidelity of stimulus representations in visual regions and increased connectivity of stimulus information between visual and dorsal attention network regions, suggesting that optimal attention facilitates both the representation and transmission of task relevant visual features (Rothlein, DeGutis, & Esterman, 2018). In assessing the relationship between image similarity of the traditional stimuli of the gradCPT and performance on the task, Rothlein, DeGutis, Germine, Wilmer, McGlinchey, and Esterman (2018) created new metrics that allow for quantification of the similarity of two scene images. These metrics are based on the concept of scene gist, or the underlying spatial layout of a scene that is predominantly captured in low frequency information (Greene & Oliva, 2009; Oliva, 2005; Oliva & Torralba, 2001) and pixel intensity that captures high-frequency position-specific information. Rothlein et al. (2018) determined that individual sensitivity to stimulus similarity on the gradCPT tends to predict better signal discrimination and lower reaction time variability. Although these studies suggest that sensitivity to the similarity of high resolution/contrast scene images affects sustained attention ability, it is unknown if similarity and sustained attention performance are influenced by declines in stimuli resolution and contrast. Reductions in image contrast and resolution have been previously discussed in terms of increasing task difficulty, but difficulty is often operationally defined in terms of behavioral performance. For tasks like the gradCPT, which involve discrimination of natural scenes, utilization of similarity metrics provides an opportunity to test if difficulty can be defined by the degree to which reductions in image quality makes a given image more similar to other images, regardless of the manner by which image degradation occurs. Image similarity measures could therefore serve as a common metric to allow for comparison between the two types of degradation and their impact on sustained attention and scene discrimination ability.

To address the questions outlined above, the current study completed a parametric investigation of how image degradation due to reductions in the resolution or contrast of stimuli impacts discrimination ability for scene images used in the gradCPT. To do this, a modified two alternative forced choice (2AFC) task was developed that presented these scene images under either dynamic (gradual onset) or static (abrupt onset) image presentation sequences to assess how the impact of image degradation on performance changes with the temporal presentation of stimuli when the same scene images are used as stimuli. It was predicted that the increased visual difficulty of gradual image presentations coupled with the rapid temporal presentation structure would increase the impact of image degradation manipulations on discrimination accuracy relative to abrupt onset presentations. These tasks were also compared against performance on the original gradCPT to determine the magnitude of performance decrements over time with disparate task parameters. In addition, the present study directly compared the impact of image degradation through reduced resolution versus contrast using measurements of gist and pixel similarity developed by Rothlein et al. (2018), to assess if similarity measures provide a common metric for understanding changes of perceptual scene properties under low vision manipulations. It was hypothesized that image degradation manipulations that increase image similarity measurements more would lead to greater deficits in discrimination ability regardless of the nature of degradation type.

## Methods

### Participants

Thirty-six naïve volunteers (29 women, mean age = 20.8 ± 2.21 years) were recruited from the Boston University and Northeastern communities. All participants reported being neurologically healthy with normal or corrected-to-normal vision and passed screening on the Freiburg Acuity and Contrast Test assessments (FrACT; Bach, 1996). This research was approved by the VA Boston Healthcare Internal Review Board, complied with American Psychological Association (APA) ethical standards, and followed the tenets of the Declaration of Helsinki. All participants provided signed informed consent before the study and were compensated $15/hour for their time.

#### Power analysis and sample size calculation

The current study used a counterbalanced order for block presentations (see below). As a result, participant sample size needed to be completed in groups of six to complete the counterbalancing sequence. Using the software program G*Power version 3.1 (Faul, Erdfelder, Lang, & Buchner, 2007) we estimated the group sizes needed to measure medium effect sizes (Cohen's *f* = 0.25) with 95% power for the 2 × 6 mixed-design ANOVAs that were the primary focus of our behavioral performance measures was 14 per group or 28 total. Thus, a sample size of 18 per group with a total sample size of 36 was chosen for the current study.

### Materials and procedure

**Figure 1.**
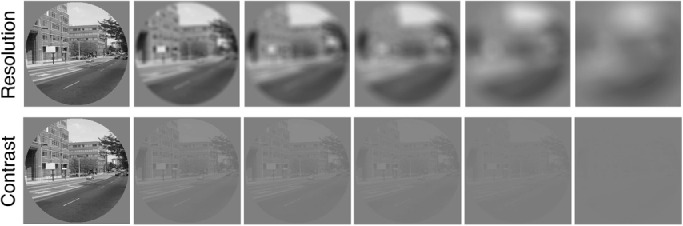
Example city image from experiment illustrating the six levels of reduced resolution using a disc blur filter (top panels) and the six levels of reduced contrast by rescaling maximum and minimum luminance of greyscale images (bottom panels).

The experiment was conducted on a 15 inch Macbook Pro (1440 × 900 screen resolution) with stimuli created using Matlab (Mathworks, Natick, MA, USA) and the Psychtoolbox (Brainard, 1997) and Palamedes (Prins & Kingdom, 2018) extensions. The following tasks used the same stimuli that were developed for the standard gradCPT task, which included 10 images of city scenes and 10 images of mountain scenes (Esterman et al., 2013). All 20 greyscale images were 300 × 300 pixels shown within a circular aperture (see Figure 1). The paradigm was modified from a go/no-go paradigm to a 2AFC task where participants were asked to discriminate between image types by pressing “z” for city images and by pressing “/” for mountain images. Unlike the traditional go/no-go gradCPT paradigm where there is a 90% probability of city images on a given trial, city and mountain images were shown with equal probability. Recent work has demonstrated that performance decrements on the gradCPT are not attributable to target frequency, with similar time-on-task decrements obtained when mountain images are presented at 50% frequency compared to 10% in the standard gradCPT design (Jun, Remington, Koutstaal, & Jiang, 2019). Given this finding and the concern that higher image degradation levels could be associated with differential changes in response patterns across individuals, namely a significant reduction in the total number of responses in blocks with highly degraded images due to shifts in criterion, a 50% target 2AFC design was chosen.

Participants were seated at a viewing distance of 50 cm from the laptop screen throughout the experiment. All participants completed four versions of the 2AFC task outlined below (a blocked task for reduced resolution and contrast, and then an adaptive task for reduced resolution and contrast). Task order was counterbalanced across participants with half of the participants completing the resolution degradation version before the low contrast versions and vice versa.

#### Blocked image degradation task

For the blocked tasks, participants were divided into two groups of 18 with one group completing the static blocked task and the other completing the dynamic blocked task. Trials in the dynamic task followed the same temporal pattern as the standard gradCPT in which images fade from one to the next every 800 ms using linear interpolation continuously for 8 minutes with no breaks provided (Esterman et al., 2013). In the static task, each trial began with a blank grey screen shown for 200 ms, followed by one of the greyscale images which remained on the screen until participants responded. Importantly, the self-paced static task was implemented to represent an optimal control condition that lacks the response time restraints and the gradual fading of the dynamic condition. This provides an upper bound of performance under image degradation using a paradigm that more closely resembles traditional perceptual discrimination tasks rather than having the temporal constraints that are associated with continuous performance tasks.

For both block types, six levels of resolution or contrast were tested within a block of trials. The six image degradation levels were determined based on pilot testing using the dynamic image presentations. Image resolution was reduced using a disc filter applied over the image with a larger radius leading to a higher defocusing of the image. Disc filter radius values used were 0, 5, 10, 15, 25, and 40 pixels (see Figure 1). This method has been shown in the literature to provide a better approximation of image distortions due to refractive errors in humans than low-pass or Gaussian filters (Strasburger, Bach, & Heinrich, 2018). Although refractive errors due to retinal disease would reduce both resolution and contrast simultaneously, the goal was to isolate these two factors to explore their impact independently of one another. To better isolate resolution changes from contrast levels, image luminance ranges were rescaled to the range of [0 to 1] after application of the disc filter.

Contrast levels were defined by the intensity range of greyscale minimums and maximums with a maximum range of 0 (black) to 1 (white) for a given image. The smaller the range between minimums and maximums, the lower the contrast of the image. The six contrast ranges tested were: 1 [0, 1], 0.164 [0.418, 0.564], 0.128 [0.436, 0.564], 0.092 [0.546, 0.454], 0.056 [0.528, 0.472], and 0.020 [0.51, 0.49]. The average log10 root mean square (RMS) values across all 20 images for these six intensity ranges was −1.47, −3.04, −3.26, −3.54, −3.98, and −4.87, respectively (see Figure 1). Movies 1 and 2 illustrate the resolution and contrast conditions for the dynamic block type.

For both block types (dynamic/static) and degradation type (resolution/contrast), each of the 20 unique images was repeated five times at a given image degradation level. Image sequence order was randomly determined for each participant with the constraint that a given image could not be presented twice in a row. Across the six resolution/contrast levels within a task, a total of 600 trials were completed. For the gradual condition, block run time was fixed at 8 minutes, whereas in the static condition, the total run time was dependent on reaction times (mean = 9.5 minutes; range = 7.0 to 13.8 minutes). Block order for the six degradation levels was determined using a six level Williams design Latin square approach to control for first-order carryover effects. Prior to beginning both the blur and contrast tasks, participants completed 120 practice trials. Here, each of the 20 images was presented once at each degradation level. During practice, resolution and contrast levels were presented in order starting with the no-degradation condition and moving down to the greatest blur/lowest contrast to help familiarize participants with the stimuli and image degradations used in the experimental blocks.

#### Adaptive image degradation task

As the blocked design required an a priori definition of the maximum image degradation (i.e. largest blur radius and lowest contrast), participants also completed an adaptive threshold version of the static presentation task for both the resolution and contrast manipulations to assess for potential group-level differences in perceptual discrimination ability. Participants began each block with a practice session of 10 trials. As in the blocked task, each trial started with a grey screen for 200 ms followed by one of the city or mountain images, which was presented until participants responded. On each trial, a random image was chosen from the 20 total images with the constraint that no image was repeated twice in a row and a total of 50 trials were completed. The adaptive task used a bestPEST (parameter estimation by sequential testing) algorithm, which does not require any prior information and assumes a constant fixed slope of the psychometric function. For this task, the bestPEST determines the disc filter radius or contrast threshold level that corresponds to a 75% accuracy rate for an individual (Bach, 1996).

#### Reference data for time-on-task decrement validation

Whereas the current dynamic version matched the standard gradCPT in almost all aspects, the present study changed both the frequency of city/mountain image presentations and the response type from the standard gradCPT. Although a recent study found that performance decrements are still observed under 50% response frequency (Jun et al., 2019), it is possible that the current changes altered the extent to which the dynamic condition tapped into sustained attention ability. To test if this was the case, we assessed whether performance deficits were observed with time on task (i.e. vigilance decrements) and if those performance decrements were similar in magnitude to those from the standard gradCPT paradigm. Data from a total of 72 participants were gathered from the study reported in Esterman, Reagan, et al. (2014) to compare time-on-task decrements between the traditional go/no-go rare-target gradCPT and the current tasks that are 2AFC with 50% stimulus frequency. This study used the same stimuli and temporal presentation structure as the dynamic condition in the current experiment, but with 10% mountain and 90% city scenes. Participants were instructed to respond to city scenes and withhold their response to mountain scenes. In Esterman, Reagan, et al. (2014), which investigated the impact of reward on participant performance, participants were assigned to reward conditions and non-reward conditions. As this study found no difference in slope estimates for accuracy and reaction time variability measures across reward conditions (i.e. no difference in the magnitude of time-on-task decrements), subjects were pooled across conditions for the purpose of our time-on-task decrement comparisons.

#### Analyses

For the blocked image degradation tasks, three performance measures were calculated. Accuracy was calculated as the proportion of correct responses across all 100 trials tested at each level of image degradation. Next, mean reaction time was calculated for trials where a correct response was made in keeping with the standard approach to assessing reaction times in the standard gradCPT task (Esterman et al., 2013). For participants in the static condition, reaction time was calculated as the time between the onset of the city/mountain image and when participants pressed one of the response buttons. For participants in the dynamic condition, reaction times were calculated using the iterative algorithm used in the standard gradCPT task (Esterman et al., 2013). Briefly, reaction time on each trial is defined relative to when the image on that trial begins to fade in from the previous image. Thus, reaction times less than 800 ms indicate the image on that trial was still fading in from the previous image, and reaction times greater than 800 ms indicate that the current trial image was in the process of being replaced by the next trial image. Given that images are presented at a fixed temporal rhythm, all button presses are recorded during completion of the task block and the iterative algorithm is used to assign reaction times after completion (see Fortenbaugh et al., 2015 for details). The third performance measure calculated for all blocks was a measure of reaction time variability (coefficient of variation [CV]). Here, reaction time variability was calculated as the CV, or the standard deviation of correct reaction times divided by the mean correct reaction time. For the adaptive classification task, image degradation levels at the last trial were taken as the threshold.

Time-on-task effects (i.e. vigilance decrements) were calculated for the four blocked image degradation conditions. As image degradation levels were intermixed in blocks of 100 trials, corresponding to 1.33-minute windows, this window size was used to calculate accuracy scores and reaction time variability for the six blocks of trials. Linear mixed effects models were then calculated, which included block time and image degradation levels as repeated fixed factors. The estimated beta parameter in this model represents the estimated slope accounting for changes in performance due to image degradation level and provided reference values that the time-on-task decrements measured in Esterman, Reagan, et al. (2014) could be compared to using one-sample *t*-tests. We note, however, that no significant differences in slopes were observed and that slopes were equivalent when the first 8-minute vigil was compared to the 10-minute slope in Esterman, Reagan, et al. (2014). For the Esterman, Reagan, et al. (2014) dataset, to ensure that similar windows of time were used across datasets, slopes for each participant were re-calculated here using linear regression on six 100 trial (1.33-minute) windows for correct omissions (COs) and reaction time variability, as rare mountain images are considered the “target” stimulus in the standard gradCPT.

As the image resolution and contrast manipulations degrade image quality in different ways, it is not possible to directly compare their impact on image discrimination ability using the native metrics (e.g. disc filter radius versus log10 RMS contrast). To assess if measurements of image similarity could provide a common metric that predict how performance is associated with changes in resolution and contrast, our final analysis utilized an approach recently developed by Rothlein et al. (2018) that characterized the similarity of a given image to all other images in the stimulus dataset. Here, the two exemplar-based similarity measures from this study were applied: cross-category pixel similarity and gist similarity. Pixel-based similarity, which is sensitive to position-specific, high-frequency commonalities across image pairs, used correlations of pixel intensity values across the grey-scaled images to determine the degree to which a given image is likely to be mistaken as an image in the other category (e.g. responding that an image is a city image when it is in fact a mountain image). For each of the 20 images in the stimulus dataset, pixel intensity values across the 300 × 300 image matrix were reshaped into a column vector and correlated with the reshaped pixel intensity values of the 10 images in the other category. That is, pixel intensity values for each city image were correlated with pixel intensity values for all 10 mountain images and vice versa, using Pearson correlation coefficients. The largest r-value was taken as the exemplar pixel similarity value for that stimulus (i.e. the r-value associated with the nearest cross-category neighbor). A single pixel similarity value was obtained for each of the 20 images in the dataset. To assess how similarity changes as a function of image degradation, the 20 images were degraded using the six blur disc filters and six contrast reduction levels in the blocked task. This allowed us to generate two 6 × 20 matrices showing the change in image pixel similarity for each image at each resolution and contrast level tested. A similar approach was used to calculate gist similarity. However, for this measure, each image was represented as a Gist descriptor vector with 512 values as defined in Rothlein et al. (2018). Briefly, each image was divided into 16 equal subsets of size 4 × 4 and 32 Gabor filters (8 orientations × 4 sizes) were applied to these subsets to obtain a measure of energy or the degree to which the oriented Gabor matched the image at that location. This measure of similarity is less sensitive to high-frequency spatial variations than the pixel similarity measure and better summarizes the spatial layout of scene images (Oliva & Torralba, 2001; Oliva & Torralba, 2006; Rothlein et al., 2018). As with the pixel similarity measure, the 512-length feature vector for each image was correlated with the 10 images from the other category and the maximum r-value was taken as the gist similarity value for that image. These calculations were run at each of the six resolution and contrast levels tested to generate the two 6 × 20 matrices showing the change in image gist similarity for each image at each resolution and contrast level tested. Defining the pixel and gist similarity values for each image allowed for assessment of how accuracy changed when images became more similar at the individual subject and group level using a common metric for both resolution and contrast manipulations.

## Results

### Participant demographics in the dynamic and static presentation conditions

Comparisons of participant demographics showed that individuals assigned to the dynamic blocked image degradation task did not differ from individuals assigned to the static blocked image degradation task in terms of gender, χ^2^(1) = 1.596, *p* = 0.402, or age, *t*(24) = 1.134, *p* = 0.265.

### Testing for time-on-task performance decrements

**Figure 2.**
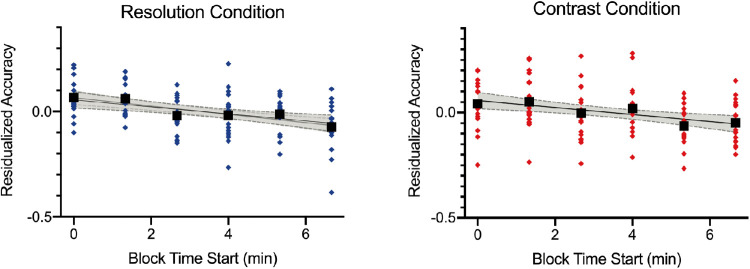
Scatterplots showing the performance decrements in accuracy over the six blocks of task in the dynamic condition after regressing out effects of image degradation level. The left panel shows performance for the resolution image degradation condition and the right panel shows performance for the contrast image degradation condition. Individual participant data are shown as blue or red diamonds. The black lines show the best-fitting linear regression model with 95% confidence intervals shaded in grey.

Performance decrements with time on task are a hallmark feature of sustained attention tasks. As the tasks used in this study were adapted from a go/no-go sustained attention paradigm with only 10% probability of a target appearance to a 2AFC discrimination task with equal probability of stimuli appearing, it was necessary to determine whether the performance decrement over time that is typically observed in the original gradCPT was also present in the 2AFC version, as would be predicted by prior work (Jun et al., 2019). To confirm this, the magnitude of the performance decrements of the traditional go/no-go gradCPT with a 10% target rate observed in Esterman, Reagan, et al. (2014) were compared to those of the current tasks. The Table 1 shows the calculated slopes for correct omissions and reaction time variability computed from the Esterman, Reagan, et al. (2014) dataset along with slope estimates for accuracy and reaction time variability in the dynamic and static blocked tasks using resolution or contrast image degradation in the current study. As seen in the Table 1, significant time-on-task effects were found in the current study, with accuracy declining over time in all four conditions (*p* values ≤ 0.008 for all). Figure 2 shows the decline in accuracy with time on task for the dynamic presentation in the resolution and contrast image degradation blocks, after residualizing out the effect of image degradation levels. Reaction times were found to become significantly more variable over time in the dynamic conditions (*p* values ≤ 0.001 for both), whereas no significant change was seen in the static conditions (*p* values > 0.73 for both). One-sample *t*-tests were calculated to determine if the slopes calculated from the Esterman, Reagan, et al. (2014) study differed from the slope parameter estimates from the current tasks. Comparison of slopes for accuracy in the current study to the slope of correct omission rates (correctly withholding responses to no-go targets) in Esterman, Reagan, et al. (2014) were completed as errors to infrequent targets occur at a higher rate than failures to respond to frequent non-target stimuli (i.e. omission errors). As seen in the Table 1, the rate at which accuracy rates declined in the current study did not differ from the rate at which correct omissions declined on the standard gradCPT for the two conditions with dynamic stimulus presentations (*p* values > 0.10 for both). With static stimulus presentations, slopes did not differ for the resolution degradation condition (*p =* 0.089), but slopes were significantly shallower for static presentations in the contrast degradation condition (*p* = 0.012). Considering changes in reaction time variability over time, results show increases in response variability in the dynamic presentation conditions that were larger than those seen in Esterman, Reagan, et al. (2014) for the resolution image degradation condition (*p* = 0.0015) or did not significantly differ from Esterman, Reagan, et al. (2014) in the contrast image degradation condition (*p* = 0.176). For the static presentation blocks, reaction time variability slopes were significantly shallower for both image degradation conditions (*p* values < 0.001 for both). Collectively, these results demonstrate significant declines in performance over time for all four conditions in the present study. Importantly, when a fixed temporal structure is utilized in the dynamic presentation condition similar to other continuous performance tasks, declines in accuracy and increases in reaction time variability were as large if not larger than those seen in the standard gradCPT paradigm over an 8-minute “vigil.”

**Table 1.** Comparisons of performance slopes in Esterman, Reagan, et al. (2014) and the current tasks are depicted based on task condition (dynamic vs. static) and image degradation condition (resolution versus contrast). Means and beta estimates are shown with standard errors. Accuracy was defined using proportion correct in the current study and correct omission rates (CO) for the data from Esterman, Reagan, et al. (2014). Note: *p < 0.05, **p < 0.01, ***p < 0.001.

	Esterman, Reagan, et al. (2014) Slopes (mean ± SE)	LME slope estimate (β ± SE)	LME slope significance (β ≠ 0)	Comparison to Esterman, Reagan, et al. (2014)	LME slope estimate (β ± SE)	LME slope significance (β ≠ 0)	Comparison to Esterman, Reagan, et al. (2014)
Dynamic		Blur			Contrast		
Accuracy	−0.0145 ± 0.003	−0.0197 ± 0.003	t(88) = −6.067, *p* < 0.001***	t(71) = 1.6635, *p* = 0.1006	−0.0166 ± 0.004	t(88) = −4.942, *p* < 0.001***	t(71) = 0.6669, *p* = 0.5070
CV	0.0214 ± 0.0008	0.0243 ± 0.006	t(88) = 4.318, *p* < 0.001***	t(71) = −3.2936, *p* = 0.0015**	0.0202 ± 0.0042	t(88) = 4.826, *p* < 0.001***	t(71) = 1.3661, *p* = 0.1762
Static		Blur			Contrast		
Accuracy	−0.0145 ± 0.003	−0.0092 ± 0.003	t(88) = −2.706, *p* = 0.008**	t(71) = −1.7258, *p* = 0.0887	−0.0065 ± 0.001	t(88) = −4.978, *p* < 0.001***	t(71) = −2.5873, *p* = 0.0117*
CV	0.0214 ± 0.0008	0.00064 ± 0.009	t(88) = 0.073, *p* = 0.942	t(71) = 23.6166, *p* < 0.001***	−0.0029 ± 0.009	t(88) = −0.338, *p* = 0.736	t(71) = 27.6882, *p* < 0.001*

### Group-level behavioral performance

**Figure 3.**
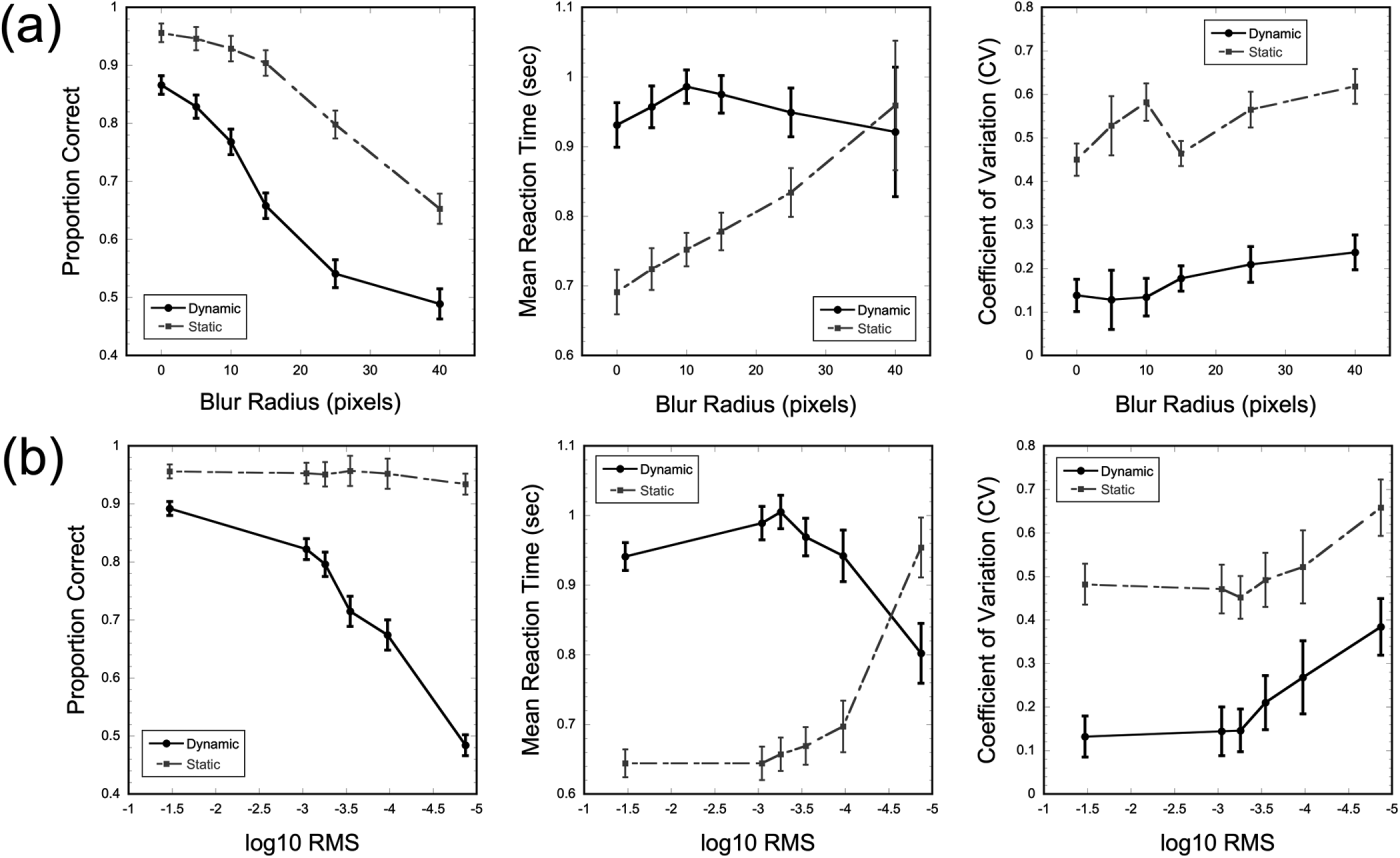
Behavioral results from blocked image degradation task. (**a**) The top panels show results from blocks where resolution was reduced used a blur disc filter. The three figures show the mean proportion correct, mean reaction time in seconds and reaction time variability calculated as the coefficient of variation as a function of blur level for participants who completed the dynamic or static conditions. (**b**) The bottom panels show results from blocks where image contrast was reduced. The three figures show the mean proportion correct, mean reaction time in seconds and the coefficient of variation as a function of contrast level calculated using log10 RMS for participants who completed the dynamic or static conditions. Error bars show ±1 SEM.

The next level of analysis examined how performance variables changed as a function of image degradation level. Figure 3 shows the group-level means for each of the three performance measures (accuracy, mean reaction time, and reaction time variability) at the six levels of image blur and reduced contrast tested. For group-level analyses of behavioral performance on the blocked image degradation task, mixed-design ANOVAs were calculated for each of the three performance measures assessed with the resolution and contrast image degradation manipulations as the dependent variable. Each ANOVA included condition (dynamic/static) as a between-subject factor and degradation level (6 levels of blur or reduced contrast) as a within-subject factor and used Greenhouse-Geisser correction when assumptions of sphericity were violated.

For the resolution image degradation conditions (see Figure 3a), there was a significant overall reduction in accuracy as the degree of blur increased, *F*(5,170) = 104.196, *p* < 0.001. Overall, accuracy was higher with static image presentation compared to the dynamic presentation, *F*(1,34) = 76.99, *p* < 0.001, and a significant interaction was seen between blur level and presentation condition, *F*(5,170) = 6.591, *p* < 0.001. For reaction time, results showed no overall main effect as the degree of blur increased, *F*(1.4,48) = 2.708, *p* = 0.093, but significantly faster reaction times with static image presentation compared to the dynamic presentation, *F*(1,34) = 13.555, *p* < 0.001. A significant interaction between blur level and presentation condition was also seen in reaction times with the greatest difference seen when no blur filter was applied and no difference in reaction times across presentation conditions with the largest blur filter, *F*(5,170) = 4.184, *p* < 0.001. Post hoc comparisons using Bonferroni correction for multiple comparisons with an adjusted critical α = 0.0083 support this pattern. Participants in the static condition were significantly faster in their correct discrimination reaction times for the four lowest blur levels (all *p* values < 0.001) but were not significantly faster in the two highest blur levels (*p* = 0.026 and *p* = 0.770, respectively). Reaction time variability generally increased as the degree of blur increased, *F*(2.9,98) = 3.887, *p* = 0.012, and was higher with static image presentation compared to the dynamic presentation, *F*(1,34) = 67.33, *p* < 0.001. Results did not show a significant interaction between blur level and presentation condition on reaction time variability, *F*(5,170) = 1.409, *p* = 0.223. For the contrast image degradation conditions (see Figure 3b), accuracy was higher with static image presentation compared to the dynamic presentation, *F*(1,34) = 96.926, *p* < 0.001. There was also a significant main effect of contrast level with accuracy dropping as contrast was reduced, *F*(5,170) = 52.687, *p* < 0.001. However, as seen in Figure 3, this overall main effect was primarily driven by reductions in accuracy in the dynamic presentation condition, which is seen in the significant interaction between contrast level and presentation condition, *F*(5,170) = 43.290, *p* < 0.001. For reaction time, results showed a general slowing of reaction times as the image contrast was reduced, *F*(2.8,95.3) = 3.159, *p* = 0.031, and faster overall reaction times with static image presentation compared to the dynamic presentation, *F*(1,34) = 54.891, *p* < 0.001. However, a significant interaction between contrast level and presentation condition was again seen with the greatest difference in reaction times when no contrast filter was applied and a reversal in reaction time patterns across presentation conditions at the lowest contrast level tested, *F*(5,170) = 35.471, *p* < 0.001. Post hoc comparisons using Bonferroni correction for multiple comparisons (critical α = 0.0083) were again calculated for reaction times. Results show that participants in the static condition were significantly faster in their correct discrimination reaction times for the five highest contrast levels (all *p* values < 0.001). Whereas participants in the static condition were numerically slower to respond at the lowest contrast condition, the difference across presentation conditions was not significantly different (*p* = 0.017). Reaction time variability also mirrored findings from the blur conditions. Here, reaction time variability increased as image contrast was reduced, *F*(5,170) = 11.053, *p* < 0.001, and was higher with static image presentation compared to the dynamic presentation, *F*(1,34) = 16.50, *p* < 0.001. The interaction between contrast level and presentation condition was not significant, *F*(5,170) = 0.489, *p* = 0.784.

**Figure 4.**
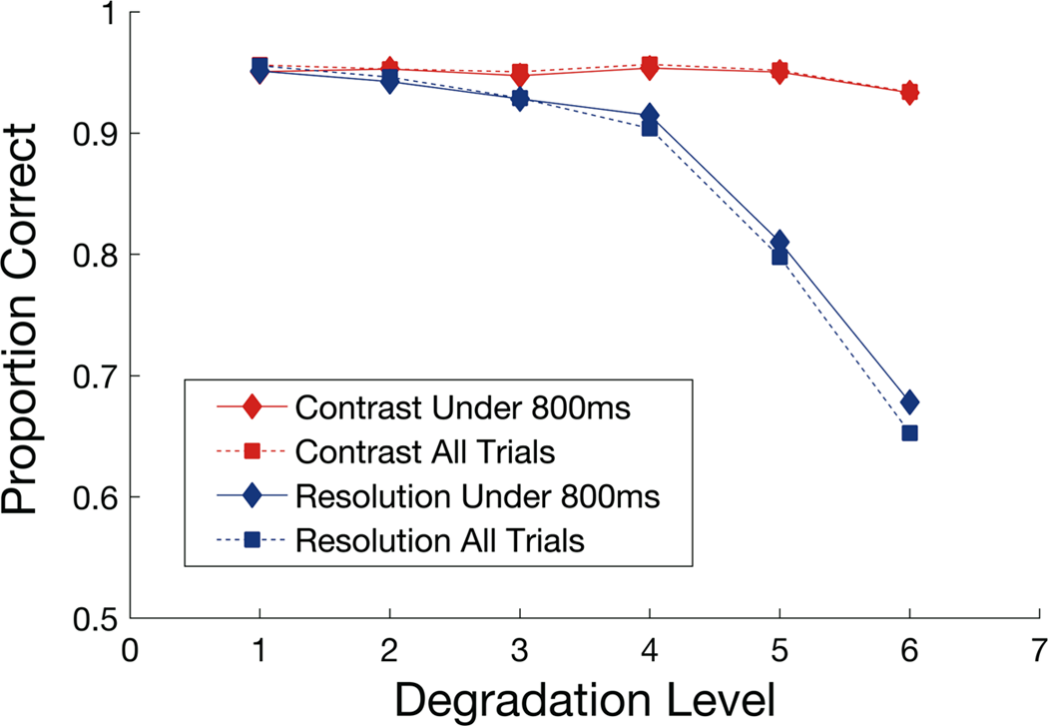
Mean proportion correct as a function of degradation level in the static conditions. The six contrast degradation levels are shown in red while the six resolution degradation levels are shown in blue (1 = no image degradation, 6 = highest image degradation block). Diamonds and solid lines show the proportion correct calculated on the subset of trials where images were shown for 800 ms or less, whereas square symbols and dashed lines show the proportion correct calculated with all trials as in Figure 3.

As images in the static condition remained on the screen until participants responded while trial durations were fixed at 800 ms in the dynamic condition, an additional qualitative analysis was completed looking at the impact of trial duration on accuracy in the static condition. Assessment of individual trial reaction times across all participants showed that with reduced resolution blocks, images were shown for longer than 800 ms on 27.8% of all trials, whereas for reduced contrast blocks, images were shown for longer than 800 ms on 18.4% of all trials. To see if higher accuracy rates in the static condition relative to the dynamic condition could be accounted for by this subset of trials where images were presented for more than 800 ms, we recalculated accuracy scores for each of the image degradation conditions excluding individual trials that presented images for longer than 800 ms. Figure 4 shows the mean accuracy rates for all trials along with the recalculated accuracy scores only considering trials where images were shown for 800 ms or less. As seen in Figure 4, the pattern of mean accuracy rates is equivalent for resolution and contrast degradation conditions, indicating that the subset of trials with longer image presentations than the gradual condition cannot account for the higher accuracy rates seen in the static presentation condition.

### Rate of change in performance across degradation levels

**Figure 5.**
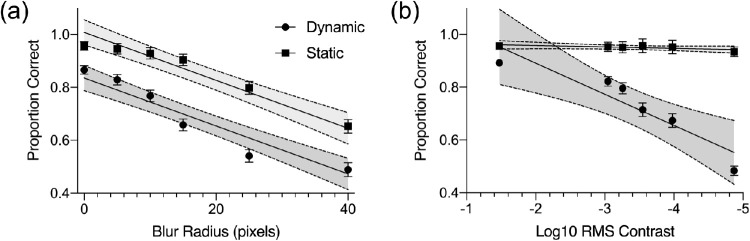
Results of hierarchical model fitting of mean proportion correct scores from the blocked image degradation task. (**a**) Results for the resolution condition showing that decreases in performance as a function of blur disc filter radius were best fit by lines with a shared slope and different intercepts across the dynamic and static conditions. (**b**) Results for the contrast condition showing the decreases in performance as a function of reductions in log10 RMS contrast were best fit by lines with different slopes and intercepts across the dynamic and static conditions. Squares and circles show mean accuracy levels at each condition with error bars showing ±1 SEM. The solid lines show the best fitting regression line with 95% CI for the regression line shaded in grey.

Whereas the ANOVAs showed a significant interaction between the degradation level and presentation conditions on the accuracy measure for both the resolution and contrast manipulations, this analysis is not well suited to address questions about rate of change when intervals are not equally spaced. To test whether performance drops at different rates with the static and dynamic image presentations as image quality is degraded, an additional hierarchical linear regression approach was also used on the group-level mean accuracy scores to test the hypothesis that performance levels drop at a faster rate in the dynamic condition than in the static condition. Here, two models were tested using GraphPad Prism (GraphPad Software, LLC version 8.4.1). The first model allowed for separate slope and intercept parameters to be fit to the group-level means for the dynamic and static conditions (i.e. performance drops at different rates). The reduced model was compared to allow for separate intercepts but required a shared slope value across the dynamic and static conditions (i.e. performance drops at the same rate). Quality of fit of the first model was compared to the reduced model using an extra sum-of-squares F-test. Figure 5 shows the results of this analysis. For the resolution condition, results from the hierarchical regression show that the alternative model with separate intercepts and slopes for the static and dynamic conditions did not provide a significantly better fit for the group-level accuracy scores, *F*(1,8) = 2.063, *p =* 0.1889. Changes in accuracy were well fit by the reduced model with two lines with a shared (global) slope parameter, with the proportion of correct responses dropping by 0.009 for every pixel increase in the blur filter radius (static condition: R^2^ = 0.94 and dynamic condition: R^2^ = 0.92). In contrast, the alternative model was found to be a significantly better fit when modeling the change in accuracy as image contrast was reduced, *F*(1,8) = 21.98, *p* = 0.0016 (static condition: R^2^ = 0.51 and dynamic condition: R^2^ = 0.86).

### Adaptive image degradation task

**Figure 6.**
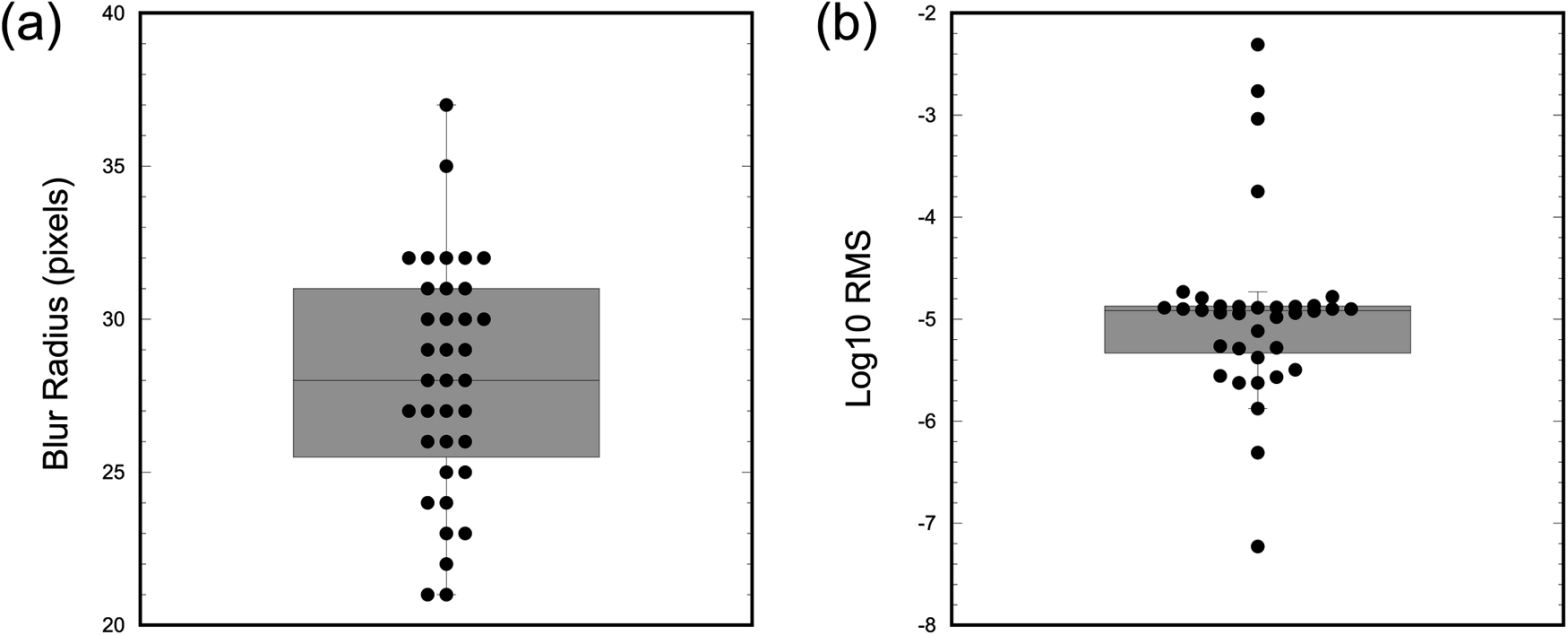
Threshold results from the adaptive image degradation task. (**a**) Box plot showing the median and interquartile range of thresholds from the resolution image degradation block indicating the radius of the disc filter in pixels. (**b**) Box plot showing the median and interquartile range of thresholds from the contrast image degradation block showing thresholds by the log10 RMS across all 20 stimulus images. For both plots, individual subject thresholds from all 36 participants are overlaid on the box plot.

The adaptive image degradation task was completed to assess for each participant the maximum degree of image degradation that could be applied under static presentation conditions where performance could be maintained at 75% accuracy. Figure 6 shows the results from the adaptive image degradation task. To test whether threshold values fell outside the range of values tested in the blocked image degradation task (max blur filter = 40-pixel radius; lowest log10 RMS contrast = −4.87), two one-sample Wilcoxon signed rank tests were calculated. Results showed that the average threshold for the resolution version of this task was significantly smaller than the largest blur disc filter radius tested (median blur filter radius: 28 pixels, z = −5.236, *p* < 0.001). In contrast, the average threshold for the contrast task was lower than the most reduced contrast condition in the blocked task (median log10 RMS = −4.92, z = −2.278, p = 0.023). While participants were split into two groups (static/dynamic) when completing the blocked image degradation task, all 36 participants completed the same adaptive image degradation task. Therefore, in order to assess for any between-group differences in performance abilities across the groups when completing the same task, Mann-Whitney *U* tests were calculated comparing thresholds on the resolution and contrast version of the adaptive image degradation task across the two groups of participants. Results of these tests showed no difference in thresholds for either the resolution or contrast tasks across the two groups of participants, indicating similar perceptual discrimination abilities at the group-level (blur: U =116, *p* = 0.152; contrast: U =120, *p* = 0.192).

### Image similarity

**Figure 7.**
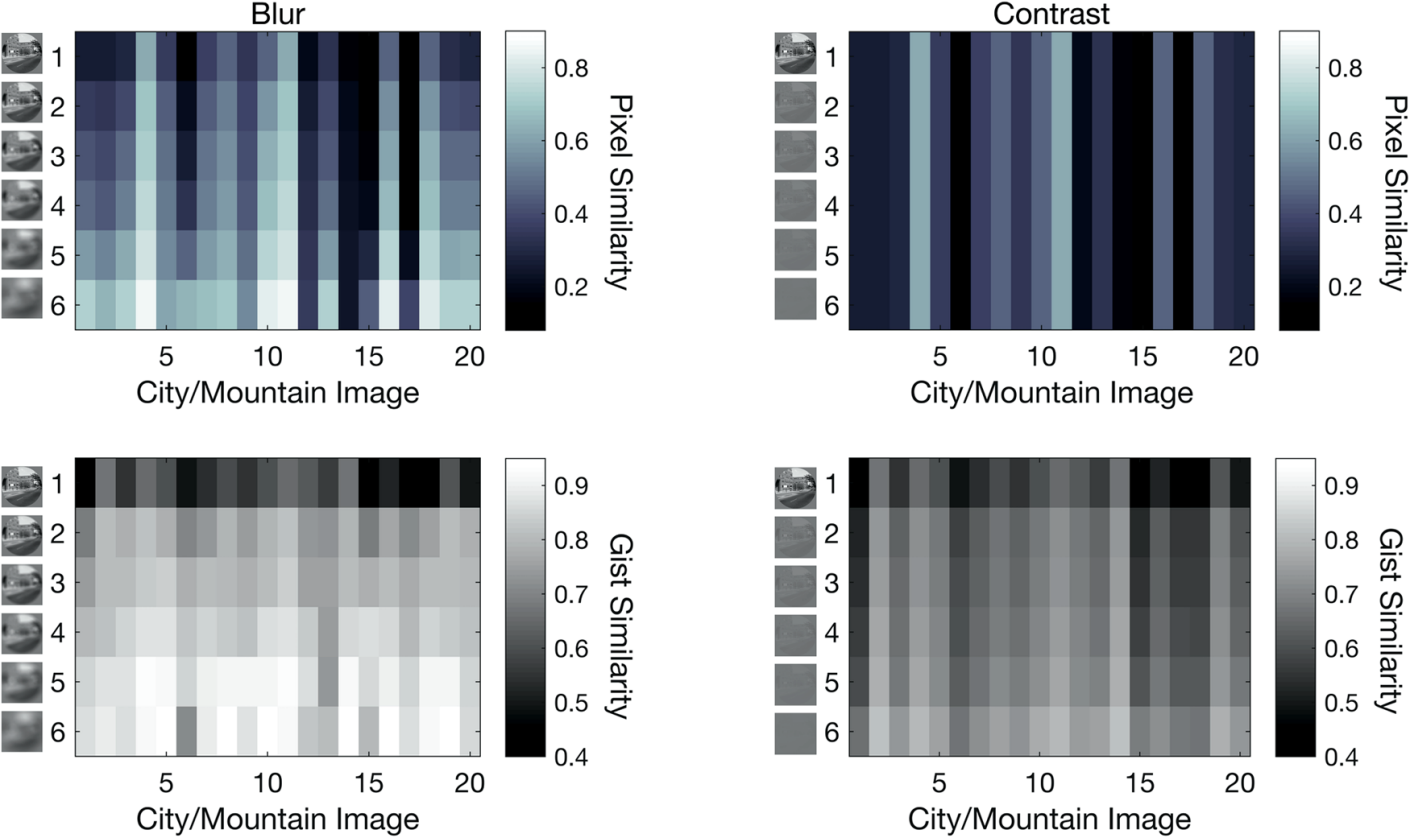
Figure illustrating the change in cross-category pixel and gist similarity measures in the reduced resolution (left panels) and reduced contrast (right panels) blocks. Pixel and gist similarity are shown for each of the 20 images used in the experiment at each of the six resolution and contrast levels tested in the blocked image degradation task. Columns 1 to 10 show the values for the city images, whereas columns 11 to 20 show the values for the mountain images.

The final analysis assessed the extent to which pixel and gist similarity measures are able to capture the relationship between image degradation and behavioral performance on the blocked task and whether these measures can provide a common metric for understanding the impact of resolution and blur on scene discrimination ability. For this analysis, images were defined based on their maximum similarity to the 10 images in the opposite category using pixel and gist similarity measures outlined in the Methods section above (Figure 7). As each image was only repeated five times at each image degradation level, for a descriptive analysis, data were concatenated across all 18 participants in each condition (dynamic/static) and proportion correct was calculated for each of the 120 images across the 90 image presentations (18 participants × 5 repeat trials) in the resolution and contrast manipulation blocks.

**Figure 8.**
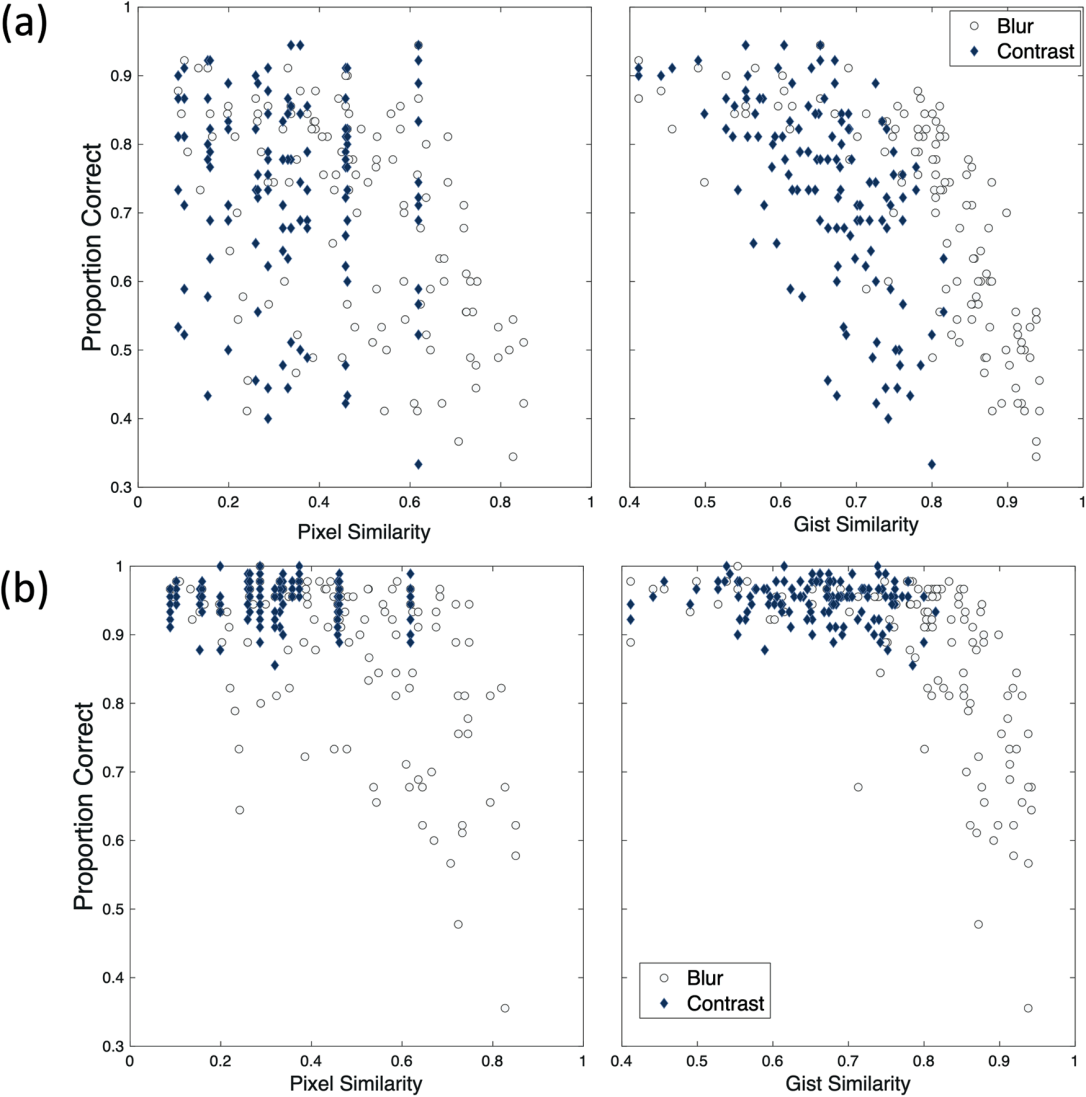
Scatterplots from image similarity analyses on (**a**) the dynamic presentation condition and (**b**) the static presentation condition. Scatterplots show the association between pixel similarity (left panels) and gist similarity (right panels) with mean accuracy scores for each image collapsed across participants. Data from the 120 images in the resolution degradation condition are shown as light grey circles while data from the 120 images in the contrast degradation condition are shown as dark blue diamonds.

Figure 8 shows the proportion correct as a function of presentation type (dynamic/static) and image similarity score. As seen in Figure 8, the gist similarity measure provides a stronger association with overall performance across both types of image degradation and both presentation types. Moreover, the results show that performance begins to drop when image similarity is lower in the dynamic presentation condition while performance remains both high and relatively unimpacted by increases in image similarity under the static image presentations until the gist similarity approaches 0.8, after which accuracy begins to rapidly decrease as images more closely resemble images in the opposite category. To provide a statistical assessment of these trends, separate linear mixed-effects models were calculated for dynamic and static presentation conditions using SPSS version 25. In each model, the mean accuracy for each image across the five repeats was included as the dependent variable. Images were combined across the resolution and contrast conditions with each participant providing accuracy scores for 240 images. Fixed factors were the pixel and gist similarity values for each image. Random effects included subject intercepts and random slopes for the pixel and gist similarity values. Results show similar trends to those seen in Figure 8. For the dynamic condition, pixel similarity was found to be negatively associated with accuracy (β = −0.1396, *p* < 0.001) as was gist similarity (β = −0.3526, *p* < 0.001). In contrast, for the static condition, gist similarity was still negatively associated with accuracy (β = −0.7826, *p* < 0.001) but no association was seen between pixel similarity and accuracy scores (β = 0.0070, *p* = 0.787). Thus, gist provides a more robust metric for associating how image degradation through reduced resolution and contrast impacts scene discrimination across both image presentation types than the pixel similarity measure.

## Discussion

The current study investigated the impact of image degradation and stimulus presentation patterns on sustained attention performance. First and foremost, this work demonstrates that the impact of image degradation on sustained attention is dependent on the way in which images are manipulated and the nature in which images are presented. Notably, accuracy decreased under both reduced resolution and contrast under complex, dynamic stimulus presentation whereas reductions in contrast were relatively stable under static stimulus presentations. Additionally, image similarity measures provide a common metric for assessing the relationship among different types of image degradation, perceptual sensitivity, and overall performance on these sustained attention tasks, suggesting that reduced resolution makes the gist of images more confusable under dynamic stimulus presentation. Moreover, as the current task response mode and stimulus frequency differ from that of the original gradCPT, we also validate that the current task is sensitive to sustained attention as performance decrements are equal to those observed in the original gradCPT.

### Greater task demands and poorer performance

Our main findings first support the notion that greater degradation of stimulus contrast and resolution leads to performance deficits. With increases in blur, accuracy declined at a similar rate under both static and dynamic presentation, but accuracy was lower overall in the dynamic stimulus presentation (see Figures 3, 5). These results highlight the deleterious impact of reduced resolution for discriminating complex natural scene images, such that even with extended viewing times and removing the fading component from the trial structure, participants were still impaired at identifying objects and forms within a given image. Conversely, reducing image contrast had little impact on performance under the static temporal pattern, whereas performance declined at a quicker rate under a gradual temporal pattern. This asymmetry suggests that, when presented with images that do not fade from one to the next, participants can successfully discriminate images even with significant reductions in contrast but fail to do so when images are presented rapidly and in a gradually fading manner. Importantly, under both contrast and resolution degradation, mean reaction times were shorter than 800 ms (trial length of the dynamic condition) in the static condition for all blocks except for the highest degraded trials, meaning images were shown for less time in the static condition on average. As seen in Figure 4, longer stimulus presentations on a subset of trials in the static condition cannot account for higher overall accuracy rates in the static condition. Rather, the pattern of results suggests that decreased perceptual saliency within the dynamic condition trials due to the complex image fading in addition to the overall image degradation manipulations likely plays a significant role by increasing stimulus ambiguity. Notably, previous literature has suggested reaction time results are driven by an initial burst of processing activation during the 240 ms of a stimulus’ display and that this period is the most critical in determining reaction time (Ulrich, Rinkenauer, & Miller, 1998). Although the different trial durations between the dynamic and static conditions pose a limitation in the interpretation of reaction time differences across the conditions, future work is needed to better characterize the impact of trial timing on overall performance. Even so, the present results suggest that time limitations alone cannot explain performance disparity between conditions. Additionally, results from the adaptive image degradation task (see Figure 6) revealed that thresholds for both the contrast and resolution degradation conditions did not significantly differ across the two groups of participants, which suggests that behavioral differences were due to the image manipulations (i.e. static versus dynamic) and not the implicit capabilities of participants in each condition.

The gradCPT (Esterman et al., 2013) is a prime example of a current sustained attention paradigm that features complex, naturalistic, gradually fading stimuli and is used in research to assess various clinical populations. The temporal dynamics of this task were designed to increase task sensitivity to fluctuations in sustained attention relative to other paradigms that use more simple stimuli and abrupt onsets that may exogenously cue attention on a trial-by-trial basis. Because complex stimuli with dynamically changing temporal patterns are becoming more common in assessments of sustained attention (Chuang, Ko, Jung, & Lin, 2014; Esterman et al., 2013; Jun & Lee, 2021; Zhang, Zhang, & Xu, 2021), this study suggests that these newer paradigms may be more sensitive to deficits in primary visual function, increasing the possibility that primary visual deficits will negatively impact performance on these tasks. From a clinical perspective, the present findings suggest that attentional failures in populations with similar low vision conditions may appear more pronounced due to reduced quality of visual input. Although this study did not feature a clinical population, the current results support the importance of considering visual status prior to attentional testing. In addition, reductions in image contrast were seen to impact performance in the dynamic condition to a far greater extent than was seen in the static condition and the results of the adaptive threshold testing showed that contrast thresholds for most participants fell outside of the range of values tested in the block design. These results suggest a need for researchers to check the testing environment when utilizing paradigms that involve complex image presentation sequences, as glare from light sources in the environment may impact image contrast (Paulsson & Sjöstrand, 1980; Rodriguez et al., 2016). Based on these findings, it is possible that suboptimal lighting conditions, whether from the environment or the computer monitor itself, may have pronounced effects on performance in dynamic testing settings regardless of the visual status of participants. With the coronavirus disease 2019 (COVID-19) pandemic there has been an accelerated interest in using telehealth for neuropsychological assessments, though work in this area has been developing for over a decade. Web-based assessments can increase clinical reach to remote populations but also reduces control of testing environments. The present results suggest that in this context, using static stimulus presentations may make for a more reliable assessment of attention on continuous performance tasks as these may be less sensitive to visual deficits or issues with testing environments that could impact image contrast.

It is important to note that response mode and stimulus frequency of the current task differs considerably from the original gradCPT in that 2AFC and 50 percent stimulus frequency have been implemented in the current experiment. Comparison of slopes between correct omissions in the original gradCPT and accuracy in the current task demonstrates statistically similar performance decrements in the dynamic condition, suggesting that the task still taps into sustained attention despite the changes in the nature of the task. This is consistent with previous work showing that altering the response frequency to equal probability results in identical performance decrements as that of the rare target version, suggesting that stimulus frequency is not the basis for changes in performance over time (Jun et al., 2019). Instead, it appears that the complexity of the stimuli coupled with short trial durations completed continuously over minutes are the main factors in inducing performance declines over time. These results are consistent with other findings demonstrating that the common cognitive operation used to engage cognitive control in go-no/go and stop signal studies is monitoring of environmental context rather than a unique cognitive operation related to motoric stopping (Chatham, Claus, Kim, Curran, Banich, & Munakata, 2012) and suggests that the critical component of tasks designed to measure sustained attention is whether they continuously engage monitoring functions over time.

### A gist framework of scene discrimination

In extension of Rothlein et al. (2018), analyses of gist and pixel similarity on degraded images suggest that degradation of resolution has the greatest impact on image similarity and that this is especially apparent in the gist-descriptor category of similarity, supporting a gist framework of naturalistic scene discrimination under low vision (see Figures 7, 8). Although contrast and resolution are considered orthogonal dimensions of early visuospatial processing (De Valois & De Valois, 1988), the relationship between visual deficits in either dimension and attentional load has been long noted in the low vision literature. Studies have found that impairments in either acuity and contrast place increasing demands on attentional resources to maintain adequate levels of performance on complex everyday tasks, such as crossing a busy street or avoiding obstacles while walking (Geruschat & Turano, 2007; Kuyk, Elliott, & Fuhr, 1998). The concept of similarity provides a common metric to investigate these two orthogonal concepts and to quantify the extent to which reductions in these domains impacts visual processing of complex scenes. Behaviorally, we observe that accuracy decreases with greater gist similarity, and that this begins to occur at a lower gist similarity level under dynamic temporal patterns compared to that of the static temporal dynamics (see Figure 8). To further support this perspective, the mixed effects model showed that gist similarity was predictive of task accuracy in both the gradual and static conditions, suggesting that as properties that affect gist become more similar, performance decreases in both temporal patterns. These findings suggest that image properties impacted by decreases in resolution that most directly related to behavioral performance were those related to perceptual gist, such as recognition of vertical or horizontal structures, or the spatial envelope related to the global degree of naturalness or ruggedness in an image. Disrupting accessibility to these distinguishing features impacted behavioral performance more than the pixel luminance similarity measure.

These analyses determine that the impact of gist similarity on performance is much more pronounced when the temporal pattern of the task increases visual load through greater stimulus presentation complexity. In the context of reduced resolution especially, we see that the holistic properties related to perceptual gist become much less recognizable and that discrimination of images is less successful with dynamic image presentation. The greater visual demands associated with dynamic temporal patterns do not appear to allow for full visual processing of semantic scene recognition, especially under decreased resolution. Because previous work has shown that incoherent jumbling of objects in a scene alters recognizability (Biederman, 1972; Biederman, 1982), it may be that reductions in resolving power in particular impact the ability to process scene-specific properties to construct a holistic semantic conclusion about complex scenes in dynamic settings. Although the ability of the human visual system to gather the gist of a scene occurs rapidly (Thorpe, Fize, & Marlot, 1996), our results show that this ability may fall short or be delayed when visual input is degraded. Whereas accuracy decreased in the dynamic condition for both resolution and contrast manipulations at higher image similarity levels, this same pattern was not seen in the static condition where accuracy was unaffected by contrast manipulations. One possible explanation for the pattern of accuracy effects is that contrast manipulations function as a veil where relevant features for image discrimination could be recovered over time in the static condition when multiple images were not overlaid, but accuracy declined under more complex temporal stimulus presentation patterns where a given trial image was only completely shown alone for 50 ms at a time. With less complexity, even if all features become more difficult to discern, the features that are extracted are still beneficial for scene discrimination across all contrast levels. Although the use of gradually fading stimuli with overlaid images may be unique to the gradCPT task, we note that utilization of complex, dynamic stimulus presentations (e.g. driving simulators and virtual environments) to increase the ecological validity of studies are increasingly being reported (Cassarino, Maisto, Esposito, Guerrero, Chan, & Setti, 2019; Chuang et al., 2014). The results from the present study suggest that both changes in visual acuity and contrast sensitivity may negatively impact performance in these types of studies given the complex and dynamic natures of visual stimuli.

### Limitations and conclusions

An important limitation to consider in this research is the meaningfulness of gist image similarity measures in other tasks that do not use naturalistic stimuli. Gist similarity measures, which focus on holistic global image properties of a scene (Greene & Oliva, 2009; Oliva & Torralba, 2006; Oliva, 2005), may not be suitable to measure similarity for SART-like tasks that use non-scene stimuli, such as letters or numbers. The analyses used are better suited to answer questions regarding complex scenes to determine the ways that individuals perceive multiple discrete scene properties as a single unit. Another important limitation is that our image degradation manipulations may not accurately capture or represent performance changes associated with deficits in visual acuity and contrast sensitivity. Assessing changes in performance across varying levels of image degradation in a within-subject design provides an opportunity to help isolate the impact of image characteristics on performance while holding the attentional ability of an observer relatively constant. Dissociating perceptual and attentional capabilities is more difficult using between-subject designs in populations where either visual, attentional, or both vision and attentional processing may be disrupted. However, directly extending conclusions about performance deficits measured with experimental manipulations of image quality to behavioral performance of individuals with deficits in acuity or contrast was not possible in the current study. Thus, an important area for future research is to assess performance on this task in individuals with confirmed acuity and contrast sensitivity deficits to determine the extent to which external image manipulations are representative of the challenges typically faced in various clinical populations.

Overall, our study highlights the importance of considering visual properties on continuous performance tasks designed to screen for sustained attention, as these tasks are often used to assess cognitive function in clinical populations where primary visual deficits occur at a higher rate than in healthy adults. Whereas the notion that clinicians ought to be sure that patients can see visual stimuli before using them to measure cognitive abilities seems like an obvious statement, a silo effect tends to exist across clinical domains such as optometry and neuropsychology. Although profound visual deficits will be readily noticed in patients and for many tasks subtle visual deficits may not impact performance, the results of the present study suggest that for CPTs, utilization of rapid trial presentations and complex visual stimuli will increase the impact of milder deficits in resolution and contrast on performance. These results point to the need for comprehensive visual screenings prior to attention testing in clinical populations and for future work to assess the potential impact of visual deficits on performance as a function of commonly used complex temporal dynamics. These results also highlight the need for communication among different departments and practices in clinical care. Because comorbidities between various psychiatric conditions and visual deficits exist, our results provide preliminary evidence that misinterpreted attention issues may be a consequence of visual problems.

## Supplementary Material

Movie 2. *Resolution Degradation.* Movie illustrating the six levels of reduced resolution tested in the blocked image degradation task. Eight images are shown at each blur level and the images are shown fading one into the next in the same manner used in the dynamic blocked image degradation condition.

Movie 2. *Contrast Degradation.* Movie illustrating the six levels of reduced contrast tested in the blocked image degradation task. Eight images are shown at each contrast level and the images are shown fading one into the next in the same manner used in the dynamic blocked image degradation condition.
